# Production of neutralizing antibody fragment variants in the cytoplasm of *E. coli* for rapid screening: SARS-CoV-2 a case study

**DOI:** 10.1038/s41598-023-31369-2

**Published:** 2023-03-16

**Authors:** Aatir A. Tungekar, Rosario Recacha, Lloyd W. Ruddock

**Affiliations:** 1 Protein and Structural Biology Research Unit, Faculty of Biochemistry and Molecular Medicine, University of Oulu, 90220 Oulu, Finland; 2 Present Address: Center for Cooperative Research in Biosciences (CIC bioGUNE), Basque Research and Technology Alliance (BRTA), Bizkaia Technology Park, Building 801A, 48160 Derio, Spain

**Keywords:** Biochemistry, Biotechnology

## Abstract

Global health challenges such as the coronavirus pandemic warrant the urgent need for a system that allows efficient production of diagnostic and therapeutic interventions. Antibody treatments against SARS-CoV-2 were developed with an unprecedented pace and this enormous progress was achieved mainly through recombinant protein production technologies combined with expeditious screening approaches. A heterologous protein production system that allows efficient soluble production of therapeutic antibody candidates against rapidly evolving variants of deadly pathogens is an important step in preparedness towards future pandemic challenges. Here, we report cost and time-effective soluble production of SARS-CoV-2 receptor binding domain (RBD) variants as well as an array of neutralizing antibody fragments (Fabs) based on Casirivimab and Imdevimab using the CyDisCo system in the cytoplasm of *E. coli*. We also report variants of the two Fabs with higher binding affinity against SARS-CoV-2 RBD and suggest this cytoplasmic production of disulfide containing antigens and antibodies can be broadly applied towards addressing future global public health threats.

## Introduction

The role of recombinant antibodies with neutralizing potential has been amply demonstrated as an important therapeutic intervention against global pandemics^1^. With the continuous emergence of multiple pathogenic variants, identifying and producing suitable antibody candidates can be a time-consuming and expensive process. Development of cost-effective heterologous protein production and rapid screening tools can help in speeding up therapeutic strategies and hence significantly contribute towards tackling present and forthcoming infectious disease outbreaks.

For instance, the pandemic caused by Severe acute respiratory syndrome coronavirus 2 (SARS-CoV-2) started in late 2019 and brought the world to a standstill. The research community has been working on the coronavirus family for decades, and while SARS-CoV-2 shares less than 80% identity to the previously characterized SARS-CoV^2^, the spike proteins on their surface bind to the same cell surface receptor Angiotensin-converting enzyme 2 (ACE-2). Multiple studies have investigated and reported the mechanisms behind the interaction of SARS-CoV-2 with ACE-2^3–6^. The spike proteins on the surface of the virion comprise of two subunits, namely S1 and S2. The S1 subunit has four sub-domains S1^A^-S1^D^, of which the S1^B^ domain encodes the receptor binding domain (RBD) which binds to the ACE-2 receptor on host cells^7^. Further characterization of the SARS-CoV-2 RBD-ACE2 complex via X-ray crystallography (PDB ID: 2AJF) revealed the receptor binding motif (RBM) which contains most of the interface residues of SARS-CoV-2 RBD that bind to ACE2^5^.

The RBD of SARS-CoV-2 has been antigenically tested in a multitude of investigations to understand antibody responses in model organisms^8,9^ which has led to its application as a candidate subunit vaccine^10^. It has been shown that the RBD can be produced recombinantly in multiple expression hosts such that it retains its native structure and function required for antigenicity^8,11,12^. The rapidly evolving nature of virulent pathogens results in the emergence of multiple variants and efficient production of the domain that targets host cell receptors offers an attractive solution for diagnostic and therapeutic screening.

In addition to studying the virus spike protein and its subunits, a significant proportion of COVID-19 related research has focused on elucidating the mechanism of action of neutralizing antibodies^13^. Many neutralizing antibodies that target the SARS-CoV-2 spike have been identified of which a large subset function by competitively inhibiting ACE2 interaction through binding to the RBD^14–16^. Antibodies that typically target the RBM portion of the RBD have been found to display a higher neutralization potency than those targeting the non-RBM portions of the RBD and the N-terminal domain (NTD)^7^. Of the antibodies tested, an IgG antibody cocktail was developed by Regeneron (REGN-CoV2) and shown to bind at different epitopes of the RBD of SARS-CoV-2 in a non-competitive manner increasing the efficacy against virus escape mutants^16,17^. REGN10933 (Casirivimab) was shown to bind to the top of the RBD effectively overlapping the ACE2 binding site while REGN10987 (Imdevimab) was shown to bind to the side of the RBD away from the REGN10933 epitope in a way to interfere with ACE2 interactions (PDB ID:6XDG)^16^. Although these full-length monoclonal antibodies are recombinantly produced in mammalian hosts, the part of the antibody that is involved in binding to the RBD i.e., antibody fragment (Fab), can be produced in alternative expression hosts in a cheaper and efficient manner for rapid screening.

*Escherichia coli* is one of the most commonly used microbial hosts used for efficient production of heterologous proteins. However, production of disulfide-bonded proteins in the reducing cytoplasm of *E. coli* requires the activity of redox catalysts provided by the CyDisCo system. This system expresses Erv1p (*S. cerevisiae*) a sulfhydryl oxidase which catalyses the oxidation of thiol groups in proteins to form disulfide bonds, and protein disulfide isomerase (PDI; *H. sapiens*) which catalyses the isomerization of non-native disulfides to their native state^18,19^. In this article, we report CyDisCo-based production of disulfide-bonded receptor binding domain of wild-type SARS-CoV-2, Alpha (B.1.1.7) and the Omicron (BA.1) variants as well as the production of REGN10933 (Casirivimab) and REGN10987 (Imdevimab) wild type Fabs in the cytoplasm of *E. coli.* In addition, we also report the production of variants of the two Fabs for rapid screening of their affinity against the RBD of SARS-CoV-2 variants. Through this approach, we have identified Fab variants that display a significant increase in their affinity towards SARS-CoV-2 RBD as compared to the wild-type Fabs confirming this approach is valid for rapid screening.

## Results

### Soluble production of the RBD of chosen SARS-CoV-2 variants

**Figure 1:**
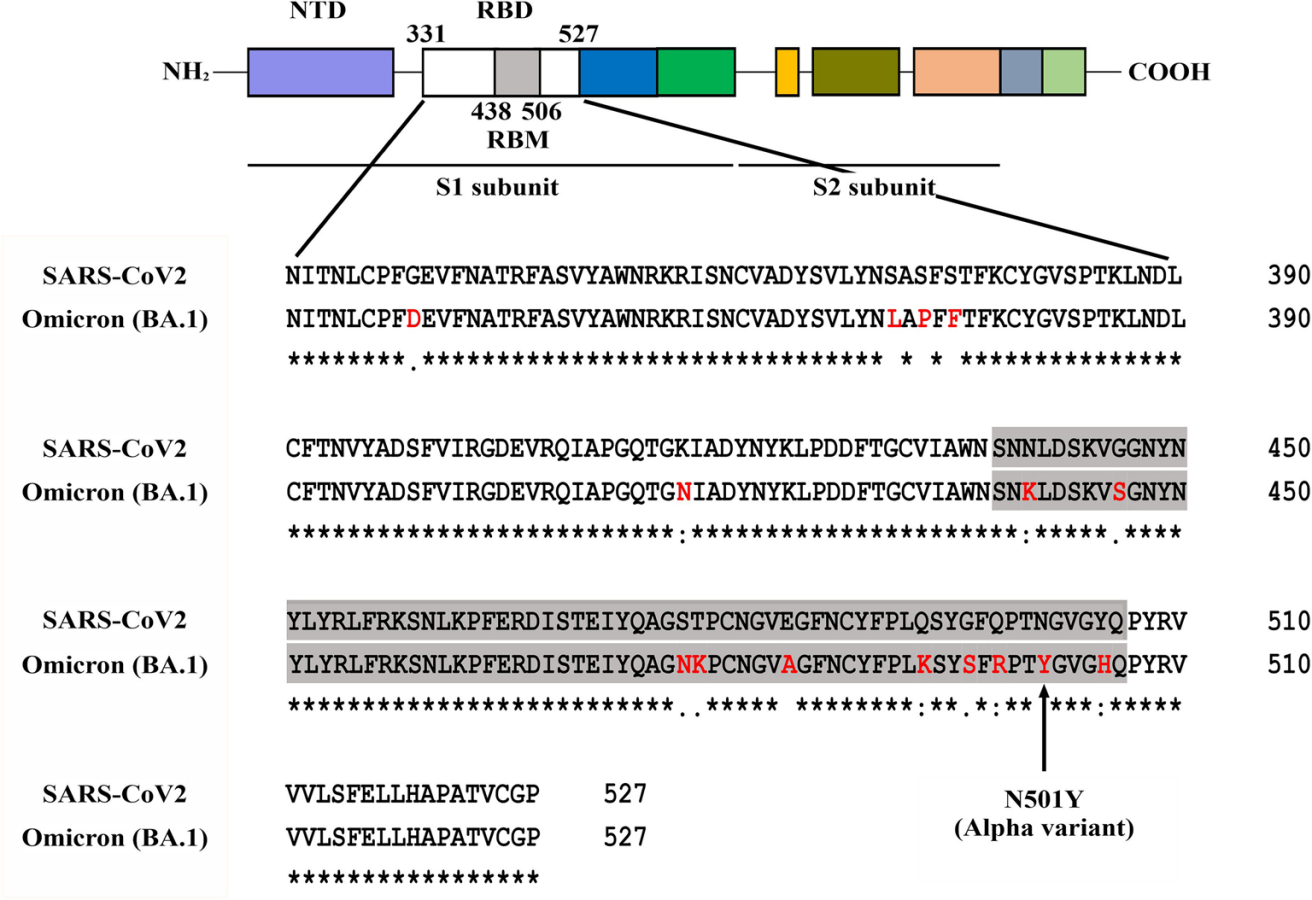
Schematic representation of the different domains of SARS-CoV-2 spike protein (*NTD* N-terminal domain, *RBD* receptor binding domain, *RBM* receptor binding motif). A graphical sequence alignment of the receptor binding domain of SARS-CoV-2 wild type and Omicron (BA.1) variant is shown. The receptor binding motif of the RBD is highlighted in grey and the mutations in the RBD between the two variants are highlighted in red. SARS-CoV-2 Alpha variant has the N501Y mutation (marked with an arrow) while the BA.1 variant has 15 mutations in the RBD with 10 of them in the RBM.

Rapid production of protein domains that play a significant role in the virulent action of pathogens can be a useful tool towards developing therapeutic strategies. The majority of these domains are disulfide-bonded proteins and their efficient production in the *E. coli* cytoplasm warrants the need of redox catalysts. The CyDisCo system employs two redox catalysts, namely Erv1p and PDI and has been used as a single polycistronic-plasmid based or dual plasmid system^19^. The combined oxidation and isomerization action of these enzymes, results in the recombinant production of natively folded disulfide-bonded proteins in the cytoplasm of *E. coli*. The CyDisCo system has previously been shown to be able to produce wild type SARS-CoV-2 RBD fused to an N-terminal maltose-binding protein (MBP) tag in a soluble and functional form in the cytoplasm of *E. coli* in low yields^20^. We chose to test the potential for CyDisCo-based production of the RBD (N331-P527) of wild type SARS-CoV-2, the Alpha variant with its principal N501Y mutation and the Omicron (BA.1) variant (Fig. 1).

**Figure 2:**
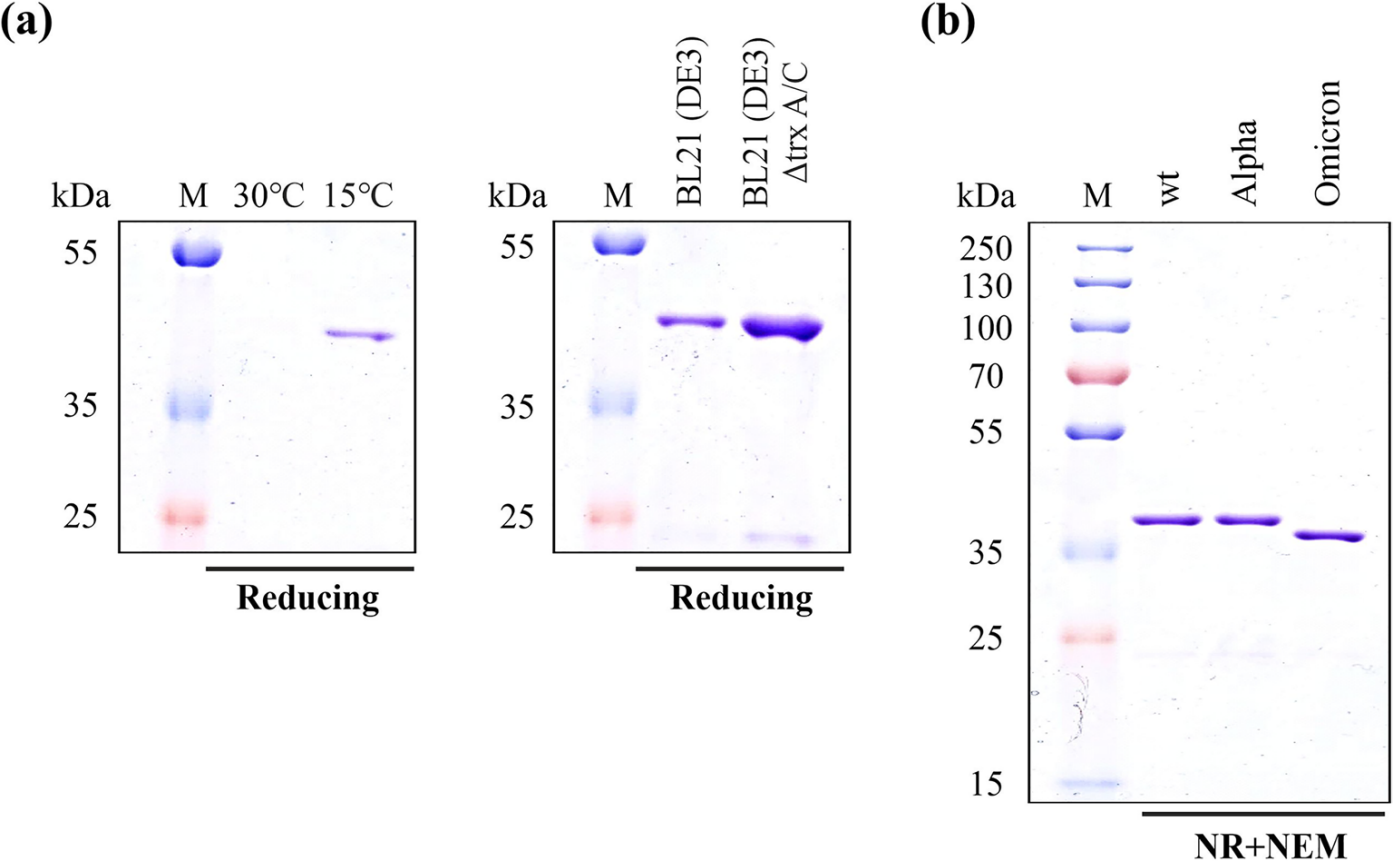
(**a**) SDS-PAGE analysis of IMAC purified mtDsbC-SARS-CoV-2 RBD wild type (wt) fusion protein under reducing conditions at two different post-induction temperatures in *E. coli* BL21 (DE3) and comparison of soluble protein obtained in two different *E. coli* strains at 15 °C. (**b**) SDS-PAGE analysis of purified mtDsbC-SARS-CoV-2 RBD fusion protein under non-reducing (NR), NEM treated conditions; (M: Protein Marker). The protein of interest was purified using a combination of IMAC, ion exchange and size-exclusion chromatography. See Supplementary Fig. S3 for the uncropped gel image.

Initial screening of the CyDisCo-based production of the wild type SARS-CoV-2 RBD in the *E. coli* cytoplasm resulted in very low amounts of soluble protein. In order to increase the amount of target protein produced, we decided to fuse the N-terminus of the RBD to a solubility enhancer tag called mtDsbC (19.7 kDa). This tag is a truncated (N81-K236) version of a naturally occurring periplasmic disulfide isomerase DsbC in *E. coli* and is catalytically inactive with its active site (CGYC) cysteines mutated (C118M, C121A). It has been screened and tested to aid the folding and solubility of a range of disulfide-bonded proteins (unpublished data). The amino acid sequence of the fusion construct has been included in Supplementary Table S1. As the solubility enhancer tag mtDsbC is fused at the N-terminus of the RBD, it does not interfere with antibody binding as it is oriented away from the receptor binding motif. This is similar to the MBP fusion tag used in the previous study^20^. The mtDsbC-RBD fusion protein was then produced using CyDisCo. Higher soluble yields were obtained when the post-induction temperature was lowered to 15 °C as compared to 30 °C (Fig. 2a). As the yields were lower than we expected, we compared the production of the mtDsbC-RBD fusion in *E. coli* BL21(DE3) and *E. coli* BL21(DE3) Δ*trxA/C*. *E.coli* has two pathways for disulfide bond reduction in the cytoplasm. One is based on the thioredoxins (TrxA and TrxC) and thioredoxin reductase (TrxB), while the other is based on glutathione and glutathione reductase. A strain with a knockout in TrxA and TrxC effectively removes one of the reducing pathways, resulting in less competition between these and the oxidizing pathways introduced with CyDisCo and hence potentially favours efficient disulfide bond formation. We observed that expression in this strain at a lower post-induction temperature resulted in comparably higher amounts of soluble fusion protein (Fig. 2a). These results demonstrate that combining the action of CyDisCo components and thioredoxin knockouts in the cytoplasm of *E. coli* can result in the enhanced soluble production of some disulfide-bonded proteins. We postulate that this is linked to the degree of exposure of disulfides in the folded state or in stable folded intermediates with TrxA/C being able to catalyse reduction to the dithiol state if one or more disulfides are exposed.

Once the construct and culture conditions were optimized, the RBD of wild type SARS-CoV-2, Alpha variant (B.1.1.7) and Omicron variant (BA.1) were produced solubly with the help of the CyDisCo system and purified using a combination of immobilized metal affinity chromatography (IMAC), ion exchange and size-exclusion chromatography (Fig. 2b). Without any further process optimization, we were able to produce 16 mg/L, 13 mg/L and 12 mg/L of purified mtDsbC-RBD fusion protein of wild type, Alpha, and Omicron variants respectively from shake-flask cultures. These purified protein yields are circa 60-fold higher than previously reported yields of wild type MBP-RBD fusion with the CyDisCo system^20^ and circa 120-fold higher than periplasmically expressed MBP-RBD (Omicron BA.1) fusion^21^. The purified proteins were then subjected to electrospray ionisation mass spectrometry (ESI–MS) analysis and were found to be natively folded with all cysteines in disulfide bonds (Supplementary Table S2). The MS analysis was also performed under denaturing conditions with N-ethylmaleimide (NEM) to evaluate the presence of any free thiols in the protein. None of the samples analysed showed an increase in mass corresponding to NEM labelling (125 Da) thereby confirming that all the cysteines were in disulfide bonds (Table 1).

**Table 1:** Molecular weight analysis of purified SARS-CoV-2 RBD wild type and variants by electrospray ionization mass spectrometry (ESI–MS) under NEM treated conditions.

Protein of interest	No. of cysteines	M_theor_ (Da)	M_exp_ (Da) (+ NEM)	Δ mass
SARS-CoV-2 RBD (wild type)	10	42,115	42,105	10
SARS-CoV-2 RBD (alpha)	10	42,164	42,154	10
SARS-CoV-2 RBD (omicron)	10	42,377	42,366	11

Formation of one disulfide bond accounts for a mass difference of 2 Da in the experimental molecular weight (M_exp_) from the theoretical molecular weight (M_theor_). No NEM binding (+ 125 Da) was observed for any of the samples analyzed suggesting the absence of free thiols.

### Soluble production of wild type Fabs

**Figure 3:**
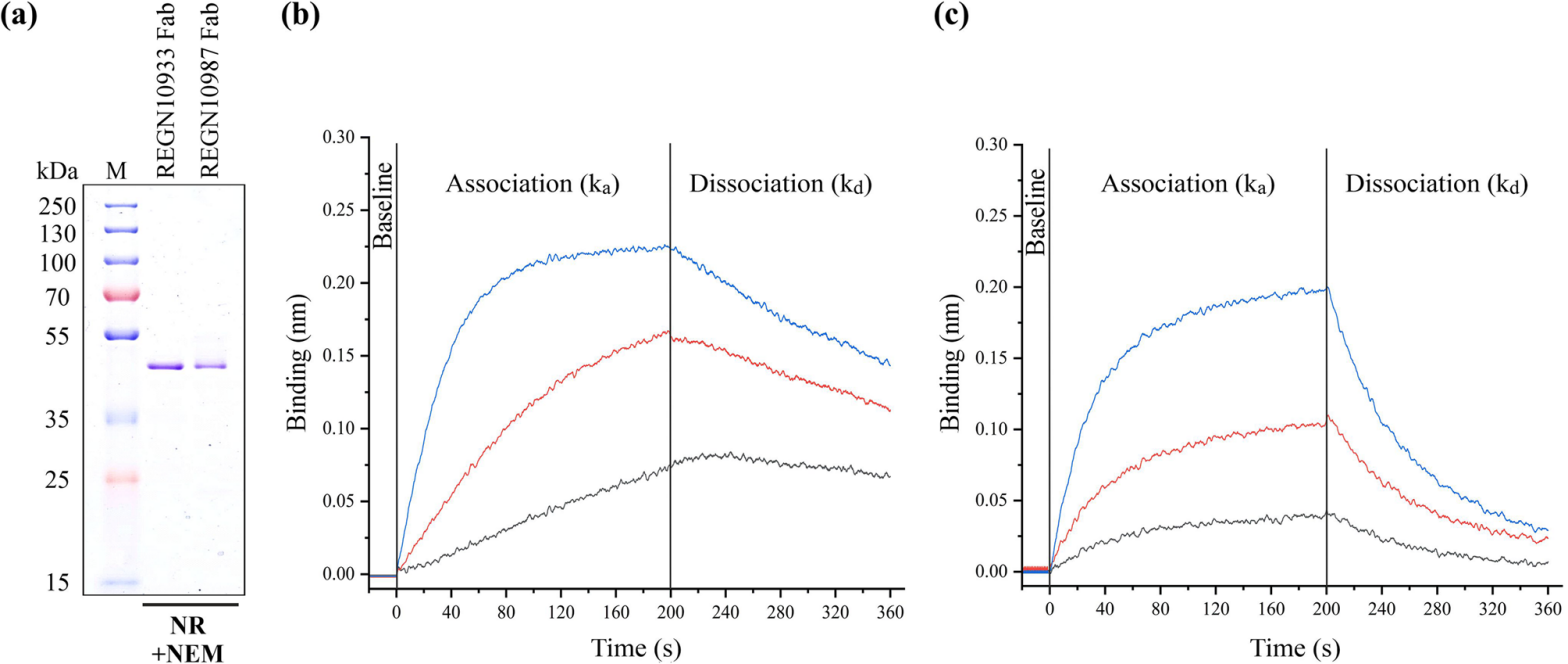
(**a**) SDS-PAGE gel image of purified REGN10933 and REGN10987 antibody fragments under non-reducing (NR), NEM treated conditions; (M: Protein Marker). The proteins of interest were purified using a combination of IMAC and size-exclusion chromatography. See Supplementary Fig. S3 for the uncropped gel image. (**b**,**c**) Biolayer Interferometry (BLI) based analysis of REGN10933 and REGN10987 Fab binding to wild type SARS-CoV-2 RBD respectively. The interaction curves show an increase in Fab-antigen association with increasing concentration of the Fabs (Grey: 3 nM, Red: 9 nM, Blue: 27 nM).

Rapid soluble production of Fabs in *E. coli* is a critical step towards developing cost and time-effective diagnostic and therapeutic interventions. All Fabs are disulfide-bonded proteins and the soluble production of these antibody formats in the *E. coli* cytoplasm can be catalysed by the CyDisCo system. The production of natively folded antibody fragments using the CyDisCo system in the cytoplasm of *E. coli* has been demonstrated earlier^22^. We tested CyDisCo-based soluble production of the wild type Fabs REGN10987 and REGN10933 in the cytoplasm of *E. coli*. In contrast to the RBD, soluble production of the two wild type Fabs was found to be optimal at 30 °C in *E. coli* BL21 (DE3) with the help of CyDisCo components. It was observed that reducing the post-induction temperature to 15 °C or using *E. coli* BL21 (DE3) Δ*trxA/C* did not improve the soluble yield. This suggests that the expression and solubility propensity is related to the nature of the target protein, and a “consensus” approach for heterologous protein production in *E. coli* is challenging to deduce. The wild type Fabs were purified using a combination of IMAC and size-exclusion chromatography (Fig. 3a) and purified yields of 10 mg/L and 37 mg/L of REGN10933 and REGN10987 Fabs respectively were obtained from shake-flask cultures. The purified proteins were subjected to ESI–MS using the same approach as aforementioned for the SARS-CoV-2 RBD. Both the wild-type Fabs were found to be folded with all cysteines in disulfide bonds (Supplementary Table S2). No increase in mass corresponding to NEM labelling (125 Da) was observed thereby confirming that all the cysteines were in disulfide bonds (Table 2).

**Table 2:** Protein yields and molecular weight analysis of purified wild-type Fabs and their variants by electrospray ionization mass spectrometry (ESI–MS) under NEM treated conditions.

Protein of interest	Purified yields (mg/L)	No. of cysteines	M_theor_ (Da)	M_exp_ (Da) (+ NEM)	Δ mass
REGN10933 Fab wild type	10	10	48,448	48,437	11
REGN10933 (T28E)	14	10	48,476	48,465	11
REGN10933 (A75S)	10	10	48,464	48,453	11
REGN10933 (M104N)	15	10	48,431	48,420	11
REGN10933 (N93Q)	10	10	48,462	48,451	11
REGN10987 Fab wild type	37	10	47,902	47,892	10
REGN10987 (L93Q)	41	10	47,917	47,907	10
REGN10987 (S95K)	48	10	47,943	47,933	10
REGN10987 (N31D)	34	10	47,903	47,893	10

Formation of one disulfide bond accounts for a mass difference of 2 Da in the experimental molecular weight (M_exp_) from the theoretical molecular weight (M_theor_). No NEM binding (+ 125 Da) was observed for any of the samples analyzed. The observed masses suggest that all cysteines are in disulfide bonds.

We next tested whether these wild type Fabs were functional and displayed binding to the wild type SARS-CoV-2 RBD using Biolayer Interferometry (BLI). Biotinylated mtDsbC-RBD fusion protein was bound as a ligand to streptavidin (SA) sensors and the Fabs were used as analytes under native conditions. We found that both of the wild type Fabs showed binding to the antigen in a concentration dependent manner (Fig. 3b,c). It was also observed that the REGN10933 Fab displays faster association (k_a_) and slower dissociation rates (k_d_) as compared to the REGN10987 Fab which implies that the former has a higher binding affinity to the wild type SARS-CoV-2 RBD. Based on BLI analysis, it was found that the REGN10933 wild type Fab binds to the SARS-CoV-2 RBD wild-type with a higher affinity (> 15 fold) as compared to the REGN10987 Fab (K_D_: 2.84 nM and 43.93 nM respectively). This is in accordance with the results obtained for their parental monoclonal IgG antibodies^16^.

### Developing Fab variants with improved affinity

The rapidly emerging variants of SARS-CoV-2 display multiple mutations in the overall spike protein of the virion with many of these located in the RBD. Some of these RBD mutations have a significant influence on the interaction with the ACE2 receptor^3,23,24^ as well as immune escape^25^. These changes necessitate rapid validation of interactions between therapeutic antibodies and the new variants as well as the establishment of new diagnostic and therapeutic antibodies which recognize the new viral variants or have higher efficacy. We hypothesized that the system used here for RBD and neutralizing Fab synthesis might also have potential for rapid screening of variants.

**Figure 4:**
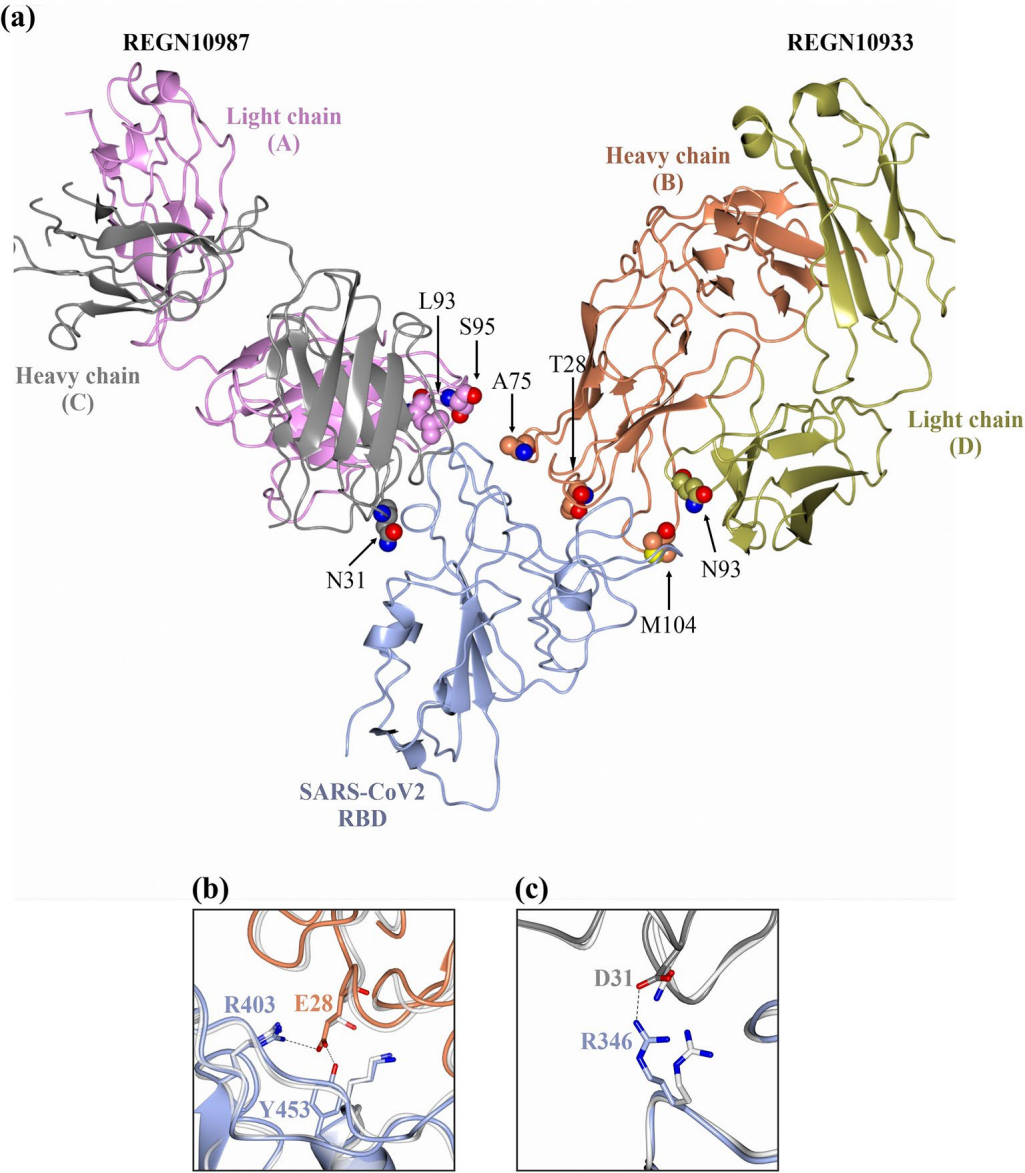
(**a**) Graphical illustration of the REGN10987 and REGN10933 Fabs bound to the SARS-CoV-2 receptor binding domain (RBD). The interface residues selected for mutation by in silico analysis have been highlighted. (**b**) Schematic representation of Thr28 of the heavy chain B (brown) mutated to Glu (E) which allows salt bridge and H-bond formation with Arg (R403) and Tyr (Y453) respectively on the SARS-CoV-2 RBD surface (blue). (**c**) Schematic representation of Asn31 of the heavy chain C (grey) mutated to Asp (D) which allows salt bridge formation with Arg (R346) on the SARS-CoV-2 RBD surface (blue). The mutations performed in silico have been overlayed against the original interactions (white backbone) and the predicted interface post energy minimization is shown. All graphical illustrations have been generated using the CCP4 Molecular Graphics program version 2.10.11 (https://www.ccp4.ac.uk/MG/)^46^.

To explore this further we made variants of the neutralizing Fabs REGN10933 and REGN10987, with mutations at the interface site between the Fab and the RBD. In silico analysis of the crystal structure of the complex^16^, along with the effects of potential mutations on local structure and antibody binding to the RBD led us to select seven mutations, split between the two Fabs so as to eliminate any Fab specific effects. Four of the selected mutations (T28E, A75S, M104N in heavy chain B and N93Q in light chain D) were in the REGN10933 Fab and three mutations (L93Q, S95K in light chain A and N31D in heavy chain C) were in the REGN10987 Fab (Fig. 4a). Several of the mutations were specifically designed to increase the affinity of the Fab for the RBD. Specifically, mutation T28E in REGN10933 heavy chain was predicted to add an additional salt bridge with R403 of the RBD and form a hydrogen-bond with Y453 (Fig. 4b), while N31D in the heavy chain of REGN10987 was predicted to form an additional salt bridge with R346 of the RBD (Fig. 4c). Mutations A75S in REGN10933 heavy chain and S95K in REGN10987 light chain were predicted to form an additional complex stabilizing hydrogen bond with the RBD (Supplementary Fig. S1). Three other mutations were made at the interfaces between Fabs and RBD with less predictable effects. N93Q in the light chain of REGN10933, M104N in the heavy chain of REGN10933 and L93Q in the light chain of REGN10987 all allow the potential for an extra hydrogen bond to be formed between the Fabs and the RBD, but none are seen in the in silico modelling (Supplementary Fig. S1). In addition, the burial of hydrophobic residues in the complex that are exposed to solvent in the free Fab is a major driving force for complex formation and hence the M104N and L93Q mutations might be expected to result in a net decrease in the affinity of the antibody for the RBD even if an additional hydrogen bond is formed.

### Soluble production of Fab variants

**Figure 5:**
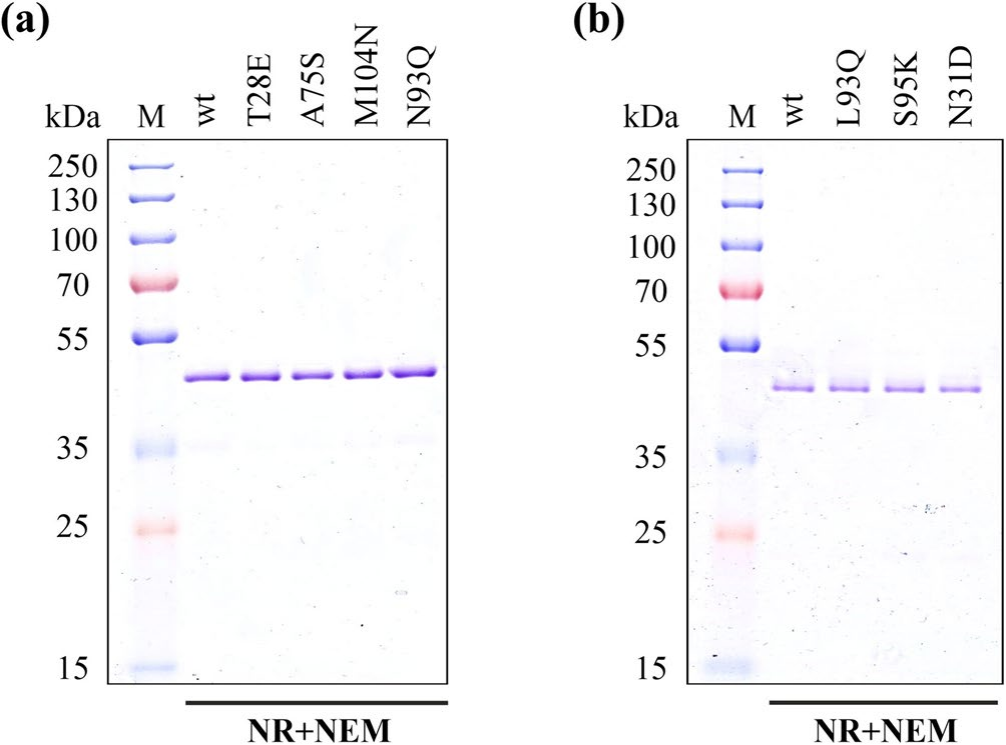
SDS-PAGE analysis of purified wild type Fabs and their variants under non-reducing (NR), NEM treated conditions (**a**). REGN10933 Fab wild type (wt) and variants (**b**). REGN10987 Fab wild type (wt) and variants (M: Protein Marker). See Supplementary Fig. S3 for the uncropped gel image.

Once the variants of the two Fabs to be investigated were chosen, we tested the soluble production of the Fab variants in the cytoplasm of *E. coli*. All seven variants of the neutralizing Fabs were produced solubly with the help of CyDisCo using the same culture conditions as that of the wild type Fabs and purified using a combination of IMAC and size-exclusion chromatography (Fig. 5). It was observed that there was no significant influence of the mutations on the yields of the target proteins as compared to the wild type proteins (Table 2). These purified proteins were also subjected to ESI–MS using the same methodology as aforementioned for the RBD. Masses obtained suggest that all the seven variants were natively produced with all the cysteines in disulfide bonds (Supplementary Table S2) and no NEM labelling was observed (Table 2).

### Effect of Fab mutations on the affinity for SARS-CoV-2 RBD variants

Employing an efficient heterologous protein production system i.e., the CyDisCo system in the *E. coli* cytoplasm allows rapid screening of the affinity of Fab variants towards the antigen in a cost-effective manner. Empirical investigation into the influence of Fab mutations on the binding affinity with the spike protein RBD was performed using Biolayer Interferometry (BLI). The affinity between the ligand (SARS-CoV-2 RBD) and analyte (Fabs) was elucidated as a function of equilibrium dissociation constant (K_D_) values based on the ratio of their dissociation (k_d_) and association (k_a_) rate constants. The K_D_ values were calculated based on a global fit analysis using three different nanomolar concentrations for each Fab.

We first tested the binding affinity of both of the wild type Fabs against the RBD of the SARS-CoV-2 Alpha variant. It was found that the REGN10933 wild type Fab was found to bind to the SARS-CoV-2 RBD of the Alpha variant with a higher affinity (> 15 fold) as compared to the REGN10987 Fab (Table 3). These results are similar to those observed with the RBD of wild type SARS-CoV-2 which implies that the N501Y mutation does not influence the binding of either of the Fabs.

**Table 3:** Biolayer Interferometry based kinetics of neutralizing antibody fragments and their variants.

Antibody fragment (Fab)	K_D_ (nM)		Effect on affinity	
	RBD SARS-CoV-2 (wild type)	RBD SARS-CoV-2 (alpha variant)	RBD SARS-CoV-2 (wild type)	RBD SARS-CoV-2 (alpha variant)
REGN10933 wild type	2.84	3.12		
REGN10933 (T28E)	< 1 pM	< 1 pM	Multifold increase	
REGN10933 (A75S)	3.22	2.75	No change	
REGN10933 (M104N)	143	119.3	~ 50-fold decrease	~ 40-fold decrease
REGN10933 (N93Q)	2.76	3.42	No change	
REGN10987 wild type	43.93	54.15		
REGN10987 (L93Q)	> 1 µM	> 1 µM	Multifold decrease	
REGN10987 (S95K)	30.53	22.78	1.4-fold increase	2.4-fold increase
REGN10987 (N31D)	17.93	13.72	2.5-fold increase	Fourfold increase

Equilibrium dissociation constant (K_D_) and the effect on affinity for the nine antibody fragments binding to receptor binding domains (RBD) of SARS-CoV-2 wild type and alpha variants are shown.

The REGN10933 Fab has been shown to bind to the spike-like loop region of the RBD such that it interferes with ACE2 binding^16^. We examined the effects of mutating Fab residues at this interface on the binding affinity for SARS-CoV-2 RBD. We hypothesize that a higher binding affinity of the REGN10933 Fab at this interface would have a significant effect on deterring ACE2 binding. The mutation T28E in heavy chain B of the REGN10933 Fab was found to result in a significant multifold increase in affinity (K_D_ < 1 pM) for SARS-CoV-2 RBD of both wild-type and alpha variants. This can be attributed to the predicted salt-bridge and H-bond formation with neighboring residues Arg403 and Tyr453 respectively on the SARS-CoV-2 RBD surface in silico (Fig. 3b). The mutations A75S in heavy chain B and N93Q in heavy chain D of the REGN10933 Fab were found to not cause any significant change in the affinity for SARS-CoV-2 RBD of both wild-type and alpha variants. However, the mutation M104N in heavy chain B of the REGN10933 Fab resulted in a significant decrease in affinity for the receptor binding domain (~ 50-fold for SARS-CoV-2 RBD wild type and ~ 40-fold for SARS-CoV-2 alpha variant). This effect could be attributed to increased hydrophilicity of the asparagine removing burial of exposed hydrophobic regions as a driving force for complex formation.

The binding site of the REGN10987 Fab on the RBD has negligible overlap with the ACE2 binding site. However, it has been shown to orient itself in a position such that interference with ACE2 binding is highly probable^16^. We screened some variants of the REGN10987 Fab to investigate the effect of the designed mutations on their binding affinity for the SARS-CoV-2 RBD. The mutation S95K in light chain A of the REGN10987 Fab was found to result in a modest increase in affinity i.e., 1.4-fold and 2.4-fold for SARS-CoV-2 RBD wild type and alpha variants respectively which can be attributed to a predicted H-bond formation with T500 of the SARS-CoV-2 RBD. In addition, the mutation N31D in heavy chain C of the REGN10987 Fab resulted in a 2.5-fold and fourfold increase in affinity for SARS-CoV-2 RBD wild type and alpha variants respectively. This increase in affinity can be attributed to a predicted salt-bridge formation with the neighboring residue Arg346 of the SARS-CoV-2 RBD (Fig. 3c). However, as we expected, the mutation L93Q in light chain A of the REGN10987 Fab resulted in a significant multifold decrease in affinity (> 1 µM) for SARS-CoV-2 RBD of both wild type and alpha variants. The equilibrium dissociation constant (K_D_) values obtained for all the interactions have been summarized in Table 3.

The wild-type Fabs and all seven variants did not display any binding to the receptor binding domain of the SARS-CoV-2 Omicron variant (Supplementary Fig. S2). As both REGN10933 and REGN10987 Fabs bind close to the receptor binding motif (RBM), it is not surprising that the ten mutations in the RBM combined with five mutations in the RBD of the Omicron variant tested seem to majorly interfere with Fab binding. This suggests that both of these antibody fragments would be rendered therapeutically inefficient against sub-variants of the Omicron lineage. These results are in accordance with the immune escape reports of Omicron variants^26,27^ as well as a neutralization sensitivity study conducted against Omicron subvariants^28^. Our findings hence validate the rapid production and screening system used in this study.

## Discussion

Recent advances in protein production and secretion capacities of microbial hosts are leading them to the forefront of therapeutic and enzyme production^29^. With the rapidly evolving nature of viruses, as seen with SARS-CoV-2 during the course of the pandemic, efficient soluble production and study of the domains involved in the virulent activity of multiple variants is a critical need. These variants possess characteristics that help them evade immune responses and antibody treatments and this can be a major threat to public health. Hence, there is an urgent need to efficiently develop, produce and screen therapeutic antibodies against newly emerging variants. *E. coli* offers many advantages for heterologous protein production, however post-translational modifications such as disulfide bond formation are a critical bottleneck. To overcome these limitations, a catalyzed disulfide forming system in the cytoplasm (CyDisCo) has been developed and employed for the production of > 500 disulfide-bonded recombinant proteins^30^.

Here, we report soluble production of the RBD of SARS-CoV-2 wild type, Alpha, and Omicron variants, fused to a solubilization tag, using the CyDisCo system in the cytoplasm of *E. coli*. While this is not the first report to produce the RBD of SARS-CoV-2 in the cytoplasm^20^ or periplasm^21^ of *E. coli*, our use of alternative media, strain and fusion partner resulted in 60–120 × higher yields than previously reported i.e., we have a significantly more efficient production system. In addition, expression of multiple variants was shown and the redox status of the purified proteins verified. With newly emerging sub-variants of the SARS-CoV-2 Omicron lineage as well as other infectious viruses, we demonstrate the potential of the CyDisCo system to be used as a rapid production system for antigenic domains of interest.

A multitude of studies in the past two years have focused on the production and testing of monoclonal antibodies either as prophylactic measures or treatment against SARS-CoV-2 infection, e.g.,^16,31–34^. While these therapeutic monoclonal antibodies are commonly produced in mammalian hosts, production of antibody fragments for rapid screening, diagnostic assays and therapeutic use can be performed more cost-effectively in microbial hosts such as *E. coli*. Antibody fragments have been most commonly produced in *E. coli* through soluble protein expression in the periplasm or in vitro refolding of inclusion bodies. However, both these methods face considerable bottlenecks in terms of productivity^35–39^. Here, we report efficient production of REGN10933 and REGN10987 neutralizing antibody fragments and in silico designed variants of both against the antigen SARS-CoV-2 RBD. We demonstrate that all the antibody fragments tested can be produced rapidly in a soluble form in the cytoplasm of *E. coli* with the help of the CyDisCo system. In addition, the wild type Fabs and variants produced were found to be functional and displayed binding to the SARS-CoV-2 RBD. We designed several mutations in silico to screen binding between the Fabs and the SARS-CoV-2 RBD and achieved > 1000-fold increase in affinity with one of the Fab variants. Our empirical results suggest that Fab variants with higher binding affinities can be rapidly developed and screened against newly emerging variants of viruses or pathogens using the methodology outlined here.

Efficient soluble production of all SARS-CoV-2 RBD and Fab variants tested in this study demonstrates the flexibility of the CyDisCo system. CyDisCo-based production of Fabs for rapid screening can be a stepping stone towards producing the most promising candidates in well-established production systems at a large scale. We believe the heterologous protein production and screening strategy outlined here provides a strong proof-of-principle and has the potential to pave the path towards cost and time-effective development of vaccines, therapeutic interventions, and diagnostic assays.

## Methods

### Cloning

Expression vectors (see Supplementary Table S3 for vectors used in this study) were constructed using standard molecular biology techniques. Gene for the SARS-CoV-2 RBD wild-type (N331-P527) and Omicron variant with a C-terminal hexahistidine tag flanked by two *BamH1* sites was synthesized codon optimized (GenScript Biotech Corp.) for *E. coli* expression. The SARS-CoV-2 RBD gene was cloned using restriction digestion and ligation using *NdeI*-*EcoRI* into a modified pET23-based vector with a pTac promoter replacing the T7 promoter^22^. To generate the mtDsbC-RBD fusion construct, the C-terminal hexahistidine tag was first removed by digestion and ligation using *BamH1*. The resulting genes were then cloned downstream of the 6X Histidine-mtDsbC gene into pGCZ141 with restriction digestion and ligation using *NdeI*-*EcoRI* to generate vectors pAAT130 and pAAT173. The cloned mtDsbC-RBD wild type fusion gene (pAAT130) was then subjected to site-directed mutagenesis (N501Y) to generate the mtDsbC-RBD fusion of the alpha variant (pAAT131). Polycistronic genes for the REGN10933 and REGN10987 wild type Fabs were synthesized codon optimized (GenScript Biotech Corp.) for *E. col*i expression. The gene for the Fab heavy chain with a C-terminal hexahistidine tag was placed downstream of the Fab light chain gene and cloned into the same backbone vector using *XbaI*-*EcoRI* to generate vectors pAAT51 and pAAT50 respectively. The variants of both Fabs were then generated using site-directed mutagenesis. Sequences of all the primers used for mutagenesis are included in Supplementary Table S4. All site-directed mutagenesis experiments were performed using the QuikChange® Site-Directed Mutagenesis Kit (Stratagene) as per manufacturers’ instructions. All plasmid vectors were purified using the E.Z.N.A Plasmid DNA Mini Kit I (Omega Bio-Tek Inc.) and DNA from agarose gels was purified using the Gene/PCR DNA Fragments Extraction Kit (GeneAid Biotech), both according to manufacturers’ guidelines. All the gene inserts in the constructed vectors were fully sequenced prior to expression tests to avoid any errors in the cloned genes.

### In silico analysis

The residues at the interface of the SARS-CoV-2 RBD and the Fabs were identified based on the structure (PDB ID: 6XDG) using the PDBePISA tool (http://pdbe.org/pisa/)^40^. Once the interface residues of the Fabs were identified, some selected residues were mutated in silico using *Coot*^41^ and the resulting complexes were energetically minimized with the geometry minimization program of the *Phenix Suite*^42^. The selected mutations were also screened using the DynaMut server (https://biosig.lab.uq.edu.au/dynamut/)^43^ to predict the effect of the mutations on protein stability and flexibility.

### Protein expression

Expression tests to screen for optimal conditions were carried out in 24-deep well plates (DWPs). The polycistronic plasmid containing the CyDisCo components Erv1p and PDI (pMJS205)^22^ along with the plasmid containing the gene of interest were used to cotransform chemically competent *E. coli* BL21(DE3) (Stratagene) or *E. coli* BL21(DE3) Δ*trxA/C*^44^ strains and allowed to grow overnight on Lysogeny Broth (LB) agar plates supplemented with appropriate antibiotics (100 µg/mL ampicillin for pET23 derivates and 35 µg/mL chloramphenicol for pLysS derivatives) at 37 °C. Selected transformants from the plates were used to inoculate 2 mL of LB media containing 2 g/L glucose and suitable antibiotics, and allowed to grow at 30 °C, 250 rpm (2.5 cm radius of gyration) in 24-DWPs covered with oxygen permeable AirOTop (Thomson) membranes for 6–8 h. These starter cultures were used to inoculate expression cultures containing 3 mL of autoclaved terrific broth autoinduction media (Formedium) supplemented with 0.8% glycerol and suitable antibiotics in a 1:100 ratio. Soluble expression of the wild type proteins (SARS-CoV-2 RBD and Fabs) in the two strains at two different post-induction temperatures 30 °C and 15 °C were compared to identify optimal expression conditions. Cells were harvested by centrifugation at 6500×*g* at 4 °C and the cell pellets were frozen at − 20 °C.

Once expression screening was completed, the main expression cultures were grown in 1 L flasks containing 100 mL culture media in each flask. These flasks were covered with oxygen permeable AirOTop (Thomson) membrane filters to ensure efficient oxygenation and incubated at 30 °C, 250 rpm for 23–24 h. For the expression of mtDsbC-SARS-CoV-2 RBD wild type and variants, once the cultures attained an optical density (600 nm) of approximately 4–5, the temperature was reduced to 15 °C and the cultures were allowed to grow for 16–18 h. Cells were harvested by centrifugation at 6500×*g* at 4 °C and the cell pellets were frozen at -20 °C.

### Protein purification

#### Cobalt-IMAC based partial purification for small-scale screening

The cell pellets from 24-DWP cultures were resuspended in 3 mL lysis buffer containing 50 mM sodium-phosphate pH 7.4, 20 μg/mL DNase, and 0.1 mg/mL egg white lysozyme. The resuspended cultures were incubated for 15 min at room temperature and frozen at − 20 °C. The cells were lysed by freeze-thawing and proteins of interest containing a hexahistidine tag were purified under native conditions using standard immobilized metal affinity chromatography (IMAC) using HisPur Cobalt Superflow Agarose (Thermo Scientific) resin following clearance of the cell lysate by centrifugation (4000 rpm, 20 min, 4 °C). For 3 mL cultures from a 24 deep well plate, 0.25 mL of resin in small Poly-Prep® gravity feed columns (Bio-Rad) was used for protein purification. The resin was washed with 2 × 5 mL of distilled water and equilibrated with 2 × 5 mL of 50 mM phosphate buffer (pH 7.4). Post sample loading, the resin was equilibrated with 2 × 2.5 mL of 50 mM phosphate buffer (pH 7.4), washed with 4 × 2.5 mL of wash buffer (50 mM sodium phosphate, 15 mM Imidazole, 0.3 M sodium chloride; pH 7.4) followed by 5 mL of equilibration buffer. The elution was carried out using 4 × 0.25 mL of elution buffer (50 mM sodium phosphate, 50 mM EDTA; pH 7.4).

#### Large-scale purification

The cell pellets were resuspended in 100 mL of lysis buffer containing 50 mM sodium phosphate pH 7.4, 20 µg/mL DNAse and 10 mM Imidazole. The cells were lysed using 18 sonication cycles (5 s pulse on, 20 s pulse off) at 70% amplitude. The cell lysate was then clarified by centrifugation at 14,500 rpm, 4 °C for 30 min and the resultant soluble fraction was filtered using a 0.45 µm membrane filter.

Large-scale purification of the mtDsbC-SARS-CoV-2 RBD wild type and variants was carried out using a combination of three chromatography steps: Immobilized Metal Affinity Chromatography (IMAC), Anion exchange chromatography (AnEx) and size exclusion chromatography (SEC). The first step was carried out with nickel IMAC for which a HiTrap™ 5 mL chelating HP column (GE Healthcare) was charged with nickel using 0.1 M Nickel chloride. The charged column was then washed by 10 column volumes (CV) of ultrapure water followed by equilibration using 20 mM sodium phosphate; pH 7.4. The soluble lysate fraction was loaded onto the Ni-IMAC column at a 2 mL/minute flow rate followed by 4 CV of equilibration buffer. Following the loading step, the column was washed with 20 mM sodium phosphate, 50 mM Imidazole, 150 mM sodium chloride; pH 7.4 followed by 4 CV of equilibration buffer. The bound proteins were eluted in a single step with 20 mM sodium phosphate, 50 mM EDTA; pH 7.4. The second step of purification was carried out using a Resource Q™ column (GE Healthcare) which was washed with 10 CV of ultrapure water and equilibrated with 20 mM Tris; pH 9.0. The IMAC eluted fraction was desalted using a 10 kDa cut off centrifugal filter (Merck), diluted 10 × with the AnEx equilibration buffer and loaded onto the column at a 2 mL/min flow rate. The bound proteins were eluted using a linear (10 CV) gradient of 20 mM Tris, 1 M NaCl; pH 9.0. The third step of purification (SEC) was carried out using HiLoad™ 16/600 Superdex™ 200 pg column (GE Healthcare). The column was washed with 1 CV of ultrapure water and equilibrated with 20 mM phosphate, 150 mM sodium chloride; pH 6.5 at a flow rate of 1 mL/minute. The AnEx eluted fractions were pooled, desalted as aforementioned, concentrated to ~ 1–1.5 mL, and used to inject the equilibrated SEC column and eluted in the same buffer.

Large-scale purification of wild type Fabs and their variants was carried using a two-step chromatography approach: immobilized metal affinity chromatography (IMAC) and anion exchange chromatography (AnEx). The IMAC step was carried out as described above, however, the elution was carried out using a linear gradient (10 CV) of 20 mM phosphate, 300 mM Imidazole, 150 mM sodium chloride; pH 7.4. The AnEx step was carried out as described above. Fractions containing the protein of interest were pooled, concentrated, flash-freezed using liquid nitrogen and stored at − 80 °C until further analysis.

SDS-PAGE analysis of reduced (β-mercaptoethanol) and non-reduced, NEM trapped samples was carried out using 12.5% SDS-PAGE gels or 4–20% Criterion™ TGX™ Precast Midi Protein Gel (Bio-Rad). All purified protein samples were subjected to SDS-PAGE analysis under non-reducing, NEM trapped conditions. The proteins samples were treated with 25 mM NEM at room temperature for 10 min prior to addition of SDS loading buffer.

### Mass spectrometry

Electrospray ionization mass spectrometry combined with liquid chromatography (LC–ESI–MS) was employed to measure the molecular weights of purified proteins using a Q Exactive Plus Mass Spectrometer (Waters). Protein samples (0.5 mg/mL) were first subjected to denaturation using 5 M guanidine hydrochloride. Post-denaturation, non-NEM trapped samples were treated with 0.5% trifluoroacetic acid (TFA) prior to analysis. NEM-trapped samples were incubated with 10 mM NEM for 10 min at room temperature and the reaction was quenched with 0.5% TFA prior to analysis. The theoretical molecular weight (M_theor_) was calculated using the amino acid sequence of the proteins using the ExPasy ProtParam tool^45^. The experimental molecular weight (M_exp_) was obtained from mass spectrometry analysis.

### Biotinylation of mtDsbC-SARS-CoV-2 RBD

Seventy µl of purified mtDsbC-SARS-CoV-2 RBD wild type (1.597 mg/mL), Alpha variant (1.270 mg/mL) and Omicron BA.1 variant (1.184 mg/mL) were mixed with 2.65 µl, 2.10 µl and 1.96 µl respectively of 1 mM EZ-Link NHS-PEG4-Biotin (Thermo Fisher Scientific) and incubated for 30 min at room temperature. The reaction was stopped by removing the excess biotin reagent using a Zeba™ Spin Desalting Column, 7 K MWCO (Thermo Fisher Scientific) as per manufacturers’ guidelines. Briefly, the column was placed in a 1.5-ml Eppendorf tube and centrifuged at 1500×*g* for one minute to remove the storage solution. The column was then washed three times with Phosphate-Buffered Saline (PBS) using the same centrifugation protocol and inserted into a new tube. The biotinylation reaction product was loaded on the top of the resin in the column and the protein was collected by centrifugation at 1500×*g* for 2 min. The protein concentration was measured at 280 nm with a NanoDrop spectrophotometer (Thermo Fisher Scientific) prior to use.

### Biolayer interferometry

In vitro interaction of the antigen mtDsbC-SARS-CoV-2 RBD wild type and variants with the two neutralizing Fabs and their variants was analyzed using an Octet RED384 instrument (ForteBio). Assays were performed at 30 °C with continuous agitation at 1000 rpm. After obtaining an initial baseline with the running buffer, 20 µg/mL of the biotinylated antigen was immobilized on Streptavidin (SA) Dip and Read™ Biosensors (ForteBio) for 600 s. All measurements were performed in 1 × PBS Kinetics Buffer (ForteBio) in 384-well microplates. Three different concentrations of each wild type Fab and their variants were parallel tested for binding to the biotinylated antigen using 10 mM sodium hydroxide, 0.75 M sodium chloride as the regeneration buffer and 1 × PBS Kinetics buffer as the neutralization buffer after each step. Data analysis was performed using a 1:1 interaction model on Octet Data Analysis High Throughput (HT) software 11.0.

## Supplementary Information

Supplementary Information.

## Data Availability

The data presented in this study is contained within the article and supplementary material. The datasets are available from the corresponding author on reasonable request.
