# Supplementary material for: Production of neutralizing antibody fragment variants in the cytoplasm of E. coli for rapid screening: SARS-CoV-2 a case study
Source: Sci Rep. 2023 Mar 16;13:4408. doi: 10.1038/s41598-023-31369-2 (PMC10019796; doi:10.1038/s41598-023-31369-2)
Supplement: Supplementary file 1 — Supplementary Information. [file 41598_2023_31369_MOESM1_ESM.pdf]

Supplementary information

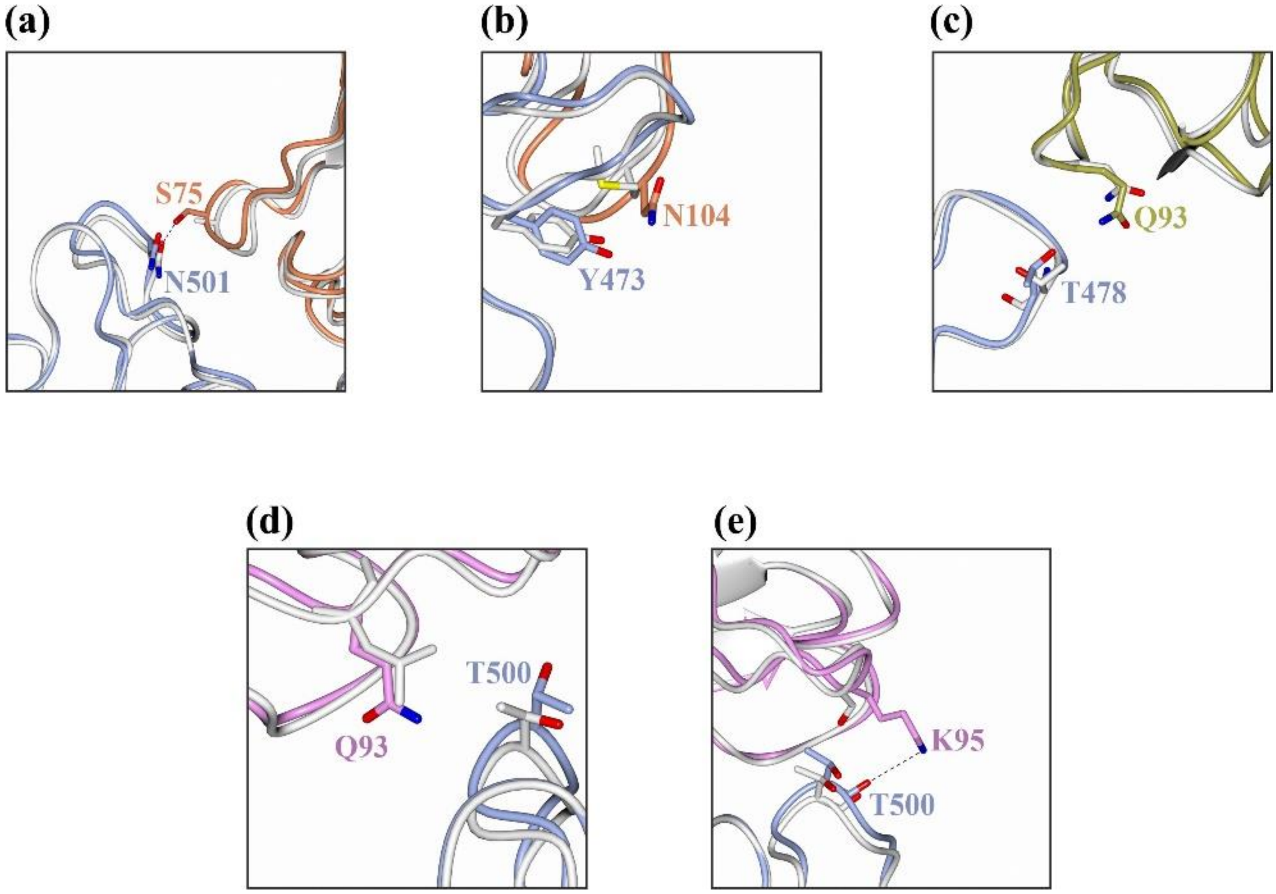

Supplementary Figure S1. Schematic representation showing mutations designed *in silico* at the interface of the Fabs and the SARS-CoV-2 RBD. The mutations performed *in silico* have been overlayed against the original interactions (white backbone) and the predicted interface post energy minimization is shown. (SARS-CoV-2 RBD: Blue, REGN10933 Heavy chain: Brown, REGN10933 Light chain: Yellow, REGN10987 Light chain: Pink). All graphical illustrations have been generated using the CCP4 Molecular Graphics program version 2.10.11 (https://www.ccp4.ac.uk/MG/) [46].

(a) (b)

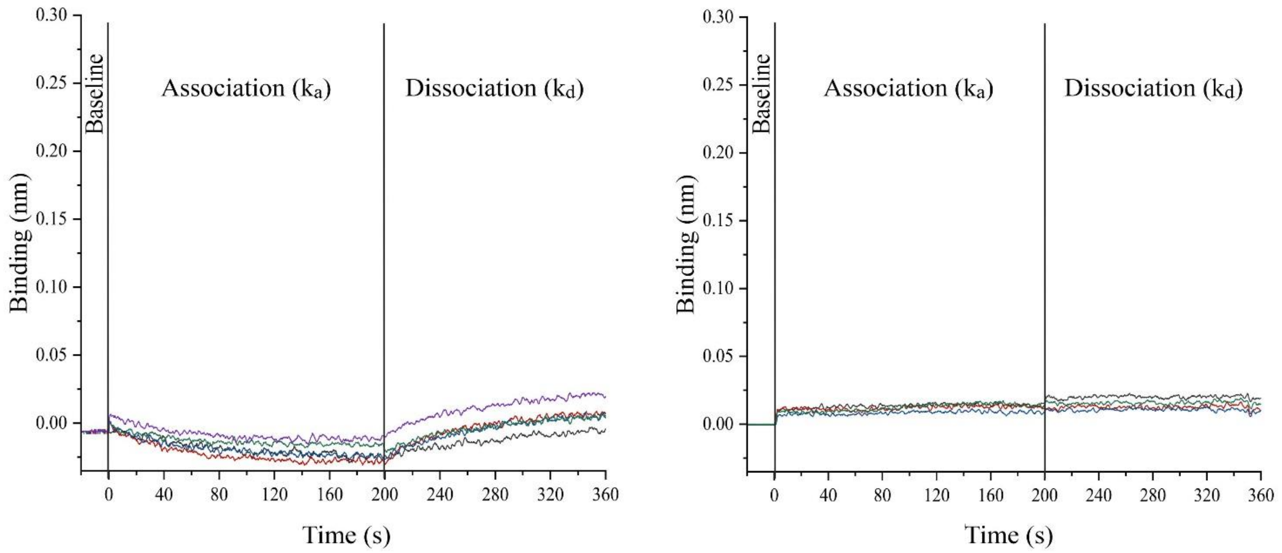

Supplementary Figure S2. Biolayer Inferometry (BLI) based analysis of wild type Fabs and their variants binding to the receptor binding domain (RBD) of the SARS-CoV-2 Omicron (BA.1) variant. Interaction curves of all Fabs tested at a concentration of 27 nm is shown. **(a).** Binding analysis of REGN10933 Fab and its variants (wild type: Black, T28E: Red, A75S: Blue, M104N: Green, N93Q: Purple). (b). Binding analysis of REGN10987 Fab and its variants (wild type: Black, L93Q: Red, S95K: Blue, N31D. Green). No binding of either the wild type Fabs or any of their variants to SARS-CoV-2 Omicron RBD was observed.

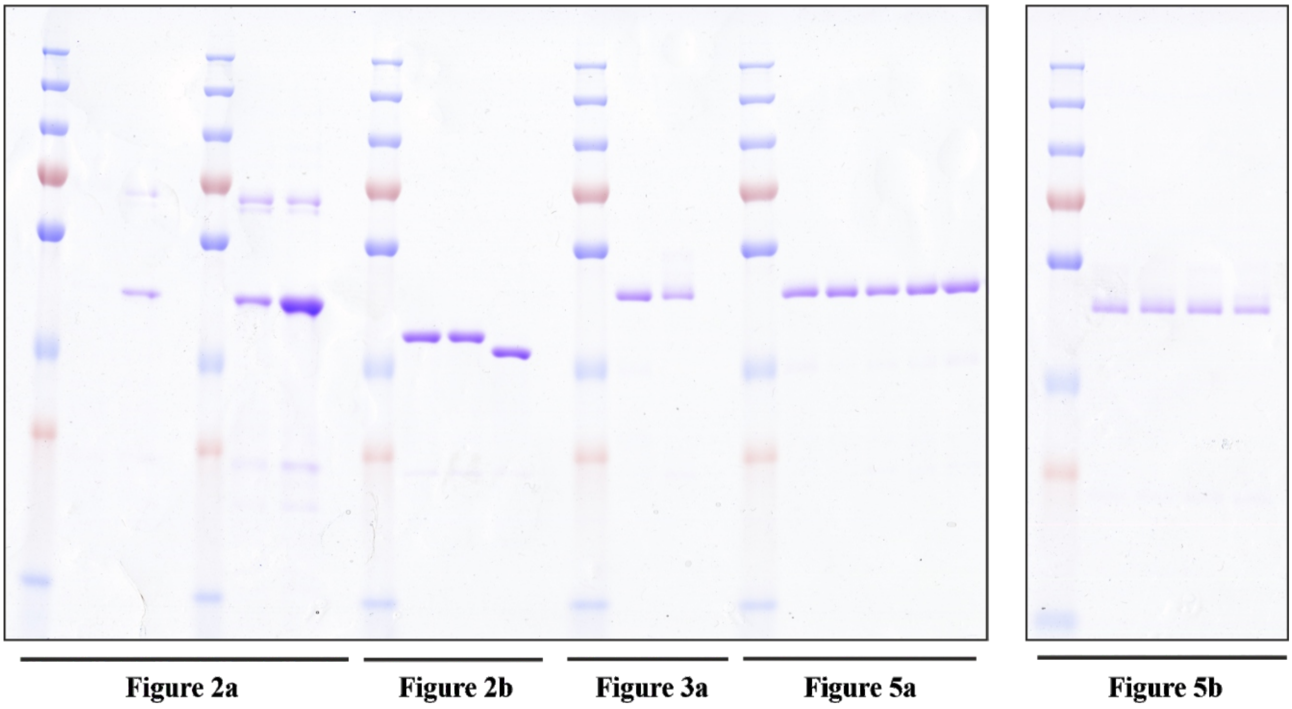

Supplementary Figure S3. Uncropped original scans of the gel images (SDS-PAGE) shown in the main article.

Supplementary Table S1. Amino acid sequence of mtDsbC-SARS-CoV-2 receptor binding domain (RBD) fusion construct.

Protein name	Sequence
mtDsbC-SARS-CoV-2 RBD (Wild type)	MGHHHHHHGSNATNKMLLKQLNALEKEMIVYKAPQEKHVITVFTDITMGYAHKLHEQMADYNALGITVRYLAFPRQGLDSDAEKEMKAIWCAKDKNKAFDDVMAGKSVAPASCDVDIADHYALGVQLGVSGTPAVVLSNGTLVPGYQPPKEMKEFLDEHQKMTSGKGGGSGSENLYFQGSHMNITNLCPFGEVFNATRFASVYAWNRKRISNCVADYSVLYNSASFSTFKCYGVSPTKLNDLCFTNVYADSFVIRGDEVRQIAPGQTGKIADYNYKLPDDFTGCVIAWNSNNLDSKVGGNYNYLYRLFRKSNLKPFERDISTEIYQAGSTPCNGVEGFNCYFPLQSYGFQPTNGVGYQPYRVVVLSFELLHAPATVCGP

Supplementary Table S2. Molecular weight analysis of purified proteins of interest by electrospray ionization mass spectrometry (ESI-MS). Formation of one disulfide bond accounts for a mass difference of 2 Da in the experimental molecular weight (M_{exp}) from the theoretical molecular weight (M_{theor}).

Protein of Interest	No. of cysteines	M_{theor} (Da)	M_{exp} (Da)	Δ mass
SARS-CoV-2 RBD (wild type)	10	42,115	42,105	10
SARS-CoV-2 RBD (Alpha)	10	42,164	42,154	10
SARS-CoV-2 RBD (Omicron)	10	42,377	42,366	11
REGN10933 Fab wild type	10	48448	48438	10
REGN10933 (T28E)	10	48476	48,466	10
REGN10933 (A75S)	10	48464	48,454	10
REGN10933 (M104N)	10	48431	48,421	10
REGN10933 (N93Q)	10	48462	48,452	10
REGN10987 Fab wild type	10	47902	47,892	8
REGN10987 (L93Q)	10	47917	47,909	8
REGN10987 (S95K)	10	47943	47,935	8
REGN10987 (N31D)	10	47903	47,894	9

Supplementary Table S3. Details of plasmid vectors used in the study

Description	Plasmid name	Reference
Truncated *E. coli* DsbC (N81-K236, C118M, C121A), N-terminal His6 tag	pGCZ141	This study
SARS-CoV-2 RBD wild type, C-terminal H6	pAAT48	This study
mtDsbC-SARS-CoV-2 RBD wild type , N-terminal His6 tag	pAAT130	This study
mtDsbC-SARS-CoV-2 RBD Alpha (N501Y), N-terminal His6 tag	pAAT131	This study
mtDsbC-SARS-CoV-2 RBD Omicron(BA.1), N and C-terminal His6 tag	pAAT172	This study
mtDsbC-SARS-CoV-2 RBD Omicron(BA.1), N-terminal His6 tag	pAAT173	This study
REGN10987 Fab wild type	pAAT50	This study
REGN10933 Fab wild type	pAAT51	This study
REGN10987 Fab (L93Q in Chain A)	pAAT104	This study
REGN10987 Fab (S95K in Chain A)	pAAT105	This study
REGN10987 Fab (N31D in Chain C)	pAAT106	This study
REGN10933 Fab (T28E in Chain B)	pAAT107	This study
REGN10933 Fab (A75S in Chain B)	pAAT108	This study
REGN10933 Fab (M104N in Chain B)	pAAT109	This study
REGN10933 Fab (N93Q in Chain D)	pAAT110	This study
CyDisCo components	pMJS205	[22]

Supplementary Table S4. Details of primers used in the study

Primer	Sequence
**Mutagenesis primers for REGN10987 Fab variants**	
L93Q - Forward	CTATTGCAACAGCCAGACCAGCATTAGCAC
L93Q - Reverse	GTGCTAATGCTGGTCTGGCTGTTGCAATAG
S95K - Forward	GCAACAGCCTGACCAAAATTAGCACCTGGGTG
S95K - Reverse	CACCCAGGTGCTAATTTTGGTCAGGCTGTTGC
N31D - Forward	CGGTTTTACCTTCAGCGATTACGCGATGTATTGGG
N31D - Reverse	CCCAATACATCGCGTAATCGCTGAAGGTAAAACCG
**Mutagenesis primers for REGN10933 Fab variants**	
T28E - Forward	GCGCGGCGAGCGGTTTTGAATTTAGCGATTACTATATG
T28E - Reverse	CATATAGTAATCGCTAAATTCAAAACCGCTCGCCGCGC
A75S - Forward	CATTAGCCGTGACAACAGCAAAAGCAGCCTGTACC
A75S - Reverse	GGTACAGGCTGCTTTTGCTGTTGTCACGGCTAATG
M104N - Forward	GTGATCGTGGTACCACCAATGTTCCGTTCGACTATTGG
M104N - Reverse	CCAATAGTCGAACGGAACATTGGTGGTACCACGATCAC
N93Q - Forward	CTATTGCCAGCAATATGATCAGCTGCCGCTGACCTTTGGTG
N93Q - Reverse	CACCAAAGGTCAGCGGCAGCTGATCATATTGCTGGCAATAG
**Mutagenesis primers for SARS-CoV-2 Alpha variant**	
N501Y - Forward	CGGCTTTCAACCGACCTATGGTGTGGGCTATCAACC
N501Y - Reverse	GGTTGATAGCCCACACCATAGGTCGGTTGAAAGCCG
